# Human Disease from Influenza A (H5N1), Thailand, 2004

**DOI:** 10.3201/eid1102.041061

**Published:** 2005-02

**Authors:** Tawee Chotpitayasunondh, Kumnuan Ungchusak, Wanna Hanshaoworakul, Supamit Chunsuthiwat, Pathom Sawanpanyalert, Rungruen Kijphati, Sorasak Lochindarat, Panida Srisan, Pongsan Suwan, Yutthasak Osotthanakorn, Tanakorn Anantasetagoon, Supornchai Kanjanawasri, Sureeporn Tanupattarachai, Jiranun Weerakul, Ruangsri Chaiwirattana, Monthira Maneerattanaporn, Rapol Poolsavatkitikool, Kulkunya Chokephaibulkit, Anucha Apisarnthanarak, Scott F. Dowell

**Affiliations:** * Queen Sirikit National Institute of Child Health, Bangkok, Thailand;; † Ministry of Public Health, Nonthaburi, Thailand;; ‡ Siriraj Hospital, Bangkok, Thailand;; § Thammasat University Hospital, Bangkok, Thailand;; ¶ International Emerging Infections Program, Nonthaburi, Thailand

**Keywords:** avian influenza, H5N1, Thailand, pneumonia, zoonosis, acute repiratory distress syndrome, season, child, research

## Abstract

Influenza A (H5N1) is endemic in poultry across much of Southeast Asia, but limited information exists on the distinctive features of the few human cases. In Thailand, we instituted nationwide surveillance and tested respiratory specimens by polymerase chain reaction and viral isolation. From January 1 to March 31, 2004, we reviewed 610 reports and identified 12 confirmed and 21 suspected cases. All 12 confirmed case-patients resided in villages that experienced abnormal chicken deaths, 9 lived in households whose backyard chickens died, and 8 reported direct contact with dead chickens. Seven were children <14 years of age. Fever preceded dyspnea by a median of 5 days, and lymphopenia significantly predicted acute respiratory distress syndrome development and death. Among hundreds of thousands of potential human cases of influenza A (H5N1) in Asia, a history of direct contact with sick poultry, young age, pneumonia and lymphopenia, and progression to acute respiratory distress syndrome should prompt specific laboratory testing for H5 influenza.

## Abstract 2

Direct contact with sick poultry, young age, pneumonia and lymphopenia, and acute respiratory distress syndrome should prompt specific laboratory testing for H5 influenza.

The 1997 outbreak of avian influenza in Hong Kong challenged the prevailing hypothesis that avian influenza viruses could infect humans only after passing through pigs or other intermediate hosts. In that outbreak, 18 persons were infected with influenza A (H5N1) virus, 6 died (*1*), and the epidemiologic and virologic evidence strongly suggested that direct contact with infected poultry was the route of transmission (*1**–**3*). All known influenza A virus subtypes that express hemagglutinins H1 to H15 and neuraminidases N1 to N9 are found in wild waterfowl (*4**,**5*), but only H1, H2, or H3 hemagglutinin subtypes had previously been known to cause human illness. Since 1997, avian outbreaks with some subtypes of influenza A viruses have been reported to cause mostly mild or inapparent infection in humans. For example, 2 mild clinical cases of H9N2 infection occurred in Hong Kong (*6*), and a large outbreak of conjunctivitis caused by H7N7 occurred in the Netherlands (*7*).

In late 2003 and early 2004, outbreaks of highly pathogenic avian influenza A (H5N1) virus infection were reported to cause lethal illness among poultry in at least 8 Asian countries (Cambodia, Indonesia, Japan, Laos, South Korea, China, Vietnam, and Thailand) (*8*). The first human cases were confirmed in Vietnam and Thailand in January 2004, and some clinical features of the first 5 Thai cases and 10 Vietnamese cases have been reported (*9**,**10*). Despite the fact that new outbreaks among poultry continued to be reported through the time of this writing (August 2004), human cases have not been recognized outside of Thailand and Vietnam. This finding may be in part because pneumonia is very common, and the distinguishing features of pneumonia caused by influenza A (H5N1) are not widely appreciated. We report the clinical details of 12 confirmed cases in Thailand and compare these with 21 suspected but unconfirmed cases and 577 reported cases that were later excluded. In addition, predictors of severe disease, pathologic features, and epidemiologic exposures are analyzed and discussed.

## Methods

### Epidemiologic Investigations

Nationwide surveillance to detect influenza A (H5N1) was initiated by the Thai Ministry of Public Health in December 2003, after outbreaks of sudden death in poultry were reported in some provinces in the central region. Under this newly established surveillance system, all patients visiting the health services with pneumonia or influenzalike illness were asked if they had been exposed to ill poultry during the preceding 7 days or had resided in an area where abnormal poultry deaths occurred during the preceding 14 days. Influenzalike illness was defined according to the World Health Organization (WHO) recommendations, which require acute fever (temperature >38.0°C) and either cough or sore throat in the absence of other diagnoses. Patients admitted with pneumonia or influenza and either of these poultry exposures were reported through the provincial public health office to the regional disease prevention and control centers and also to Bureau of Epidemiology at the Ministry of Public Health. Throat or nasopharyngeal swabs and serum samples were collected for viral study at the Thai National Institute of Health, Department of Medical Sciences. Staff members from the provincial health office visited family members to confirm history of exposure and assess the household environment.

Patients with confirmed cases of H5N1 were defined as patients reported to the system who had laboratory evidence of influenza A (H5N1) infection. Suspected case-patients were defined as patients with reported exposure to ill poultry and severe pneumonia, or patients with exposure and laboratory evidence of influenza A infection not confirmed as H5N1. Excluded case-patients were all remaining patients reported through the system who did not meet the exposure criteria or who lacked laboratory evidence of influenza A (H5N1) infection, including those with infections caused by influenza A H3 or H1, as well as other laboratory-confirmed pneumonia pathogens.

We performed comparisons of dichotomous variables by using chi-square or Fisher exact tests, as appropriate, and *t* tests for continuous variables that were normally distributed, or Wilcoxon rank-sum tests for other continuous variables. We considered p values of <0.05 to be significant.

### Laboratory Investigations

Respiratory specimens (including nasopharyngeal aspirates, nasopharyngeal swabs, nasal swabs, or throat swabs) were collected and stored in viral transport medium. Blood cultures were obtained from all patients on admission, and serum samples for mycoplasma titer and cold agglutinin testing were obtained when available. Paired serum samples taken at least 14 days apart, if available, were collected for serologic confirmation of H5N1 infection. An adequate sample was defined as any of the above respiratory specimens collected from day 2 to day 14 after onset of fever.

All specimens were submitted for testing at the National Institute of Health of Thailand, except 1, which was tested at Virology Laboratory at Siriraj Hospital, Mahidol University. Methods used for H5 identification were in accordance with those recommended by the WHO reference laboratories for influenza (*11*). Specifically, specimens in transport medium were tested by reverse transcription–polymerase chain reaction (RT-PCR) to detect nucleic acids of influenza A and B and injected onto a Madin-Darby canine kidney (MDCK) cell monolayer for viral isolation. Nasopharyngeal aspirates were agitated and centrifuged to separate the epithelial cells. Sediments of epithelial cells were tested for influenza A and B by immunofluorescence assay (IFA) with specific monoclonal antibodies. Specimens positive for influenza A were further tested for subtypes H1, H3, and H5 with specific monoclonal antibodies. The supernatant was tested by RT-PCR and viral isolation for the other types of specimens (*12*).

Specimens positive for influenza A by RT-PCR were further tested for subtypes H1, H3, and H5 by using specific primer sets. The H5-specific primer set was as follows: H5-1 GCC ATT CCA CAA CAT ACA CCC, and H5-2 TAA ATT CTC TAT CCT CCT TTC CAA, with an expected product size of 358 bp (*12**,**13*). If results were negative for all subtypes or positive for H5, they were confirmed by real-time RT-PCR using primer/probe H5 as follows: InfA_TH5_A, InfA_TH5_F, InfA_TH_Ic, and InfA_TH5_f1 (*14*). For viral isolation, if a cytopathic effect was observed, IFA was performed to identify the virus in infected cell cultures by using specific monoclonal antibodies to H1, H3, and H5. If a cytopathic effect was not observed in the first passage, the culture medium passaged in MDCK for a second time. If no cytopathic effect occurred, the negative cell culture was confirmed by IFA with pooled viral monoclonal antibodies.

Specimens were considered positive for avian influenza virus if the viral culture was positive and was confirmed by IFA with H5-specific monoclonal antibody provided by the WHO, if epithelial cells in clinical specimens were IFA positive for H5, or if the RT-PCR was positive with H5 specific primers (RT-PCR or real-time RT-PCR). A specimen was negative for avian influenza virus if IFA, RT-PCR or real-time RT-PCR, and viral isolation (second passage) were negative.

### Clinical Investigations

All potential case-patients reported through the surveillance system needed basic demographic, exposure, and clinical information recorded, as well as specimens submitted, for the purpose of case classification. Patients with suspected cases were reviewed in more detail by telephone or written correspondence with the attending physician. Laboratory-confirmed case-patients had a thorough review with standardized forms of all medical records, chest radiographs, and laboratory data by the attending physicians.

Respiratory failure was defined as requiring ventilatory support and cardiac failure as requiring inotropic drug support. Liver dysfunction was diagnosed when serum aspartate aminotransferase (AST) or alamin aminotransferase (ALT) was >8 times the upper limit of normal. Renal dysfunction was diagnosed when serum creatinine was >1.5 mg/dL. Bone marrow dysfunction was diagnosed when all 3 of the cell lines in the peripheral blood (erythrocytes, leukocytes, and platelets) were below the lower limit of normal. Leukopenia was defined as a total leukocyte count below the following age-specific cutoffs; 1–3 years <6,000, 4–7 years <5,500, and >8 years <4,500 cells/mm^3^. Lymphopenia was defined as an absolute lymphocyte count <1,500 cells/mm^3^, and thrombocytopenia was defined as a platelet count <150,000/mm^3^ (*15*).

The attending radiologist classified chest radiograph findings as normal, interstitial infiltrates, lobar infiltrates, or combinations of these by using standard criteria. Acute respiratory distress syndrome (ARDS) was defined when clinical deterioration was associated with chest radiographs showing diffuse bilateral infiltrates accompanied by severe arterial hypoxemia.

## Results

**Figure 1:**
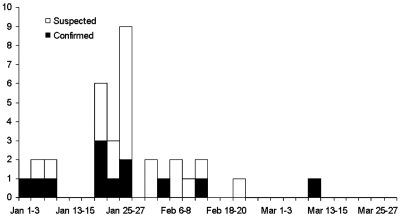
Epidemic curve showing the dates of onset for 12 confirmed and 21 suspected human cases of avian influenza A (H5N1) infection, Thailand, 2004.

From January 1 to March 31, 2004, a total of 610 cases were reported from 67 of 76 provinces in Thailand. After thorough review of the clinical, epidemiologic, and laboratory findings, we identified 12 confirmed and 21 suspected cases. The onset of illness of the first confirmed case was on January 3, and the last was on March 2 (Figure 1). A total of 577 cases were excluded, including 38 who had positive RT-PCR tests for influenza A (H3) infection, 48 seropositive for *Mycoplasma pneumoniae*, and 10 for *Chlamydophila pneumoniae*.

Table 1 compares characteristics of patients with confirmed, suspected, and excluded cases. Confirmed case-patients tended to be younger than suspected case-patients and more often had fatal disease than excluded patients (p < 0.0001). Reported poultry exposure was similar in all groups, but all confirmed patients had an adequate laboratory specimen, whereas 10% of suspected patients and 19% of excluded patients did not. All patients with an adequate laboratory specimen had testing completed.

**Table 1:** Characteristics of 12 confirmed, 21 suspected, and 577 excluded human cases of avian influenza A (H5N1) in Thailand, 2004

Characteristic	Confirmed	Suspected	Excluded
No.	12	21	577
Median age (y) (range)	12 (2–58)	33 (1–67)	12 (1–92)
Sex (% male)	67	71	59
Poultry contact (%)	58	52	48
Adequate* specimen (%)	100	90	81
Death (%)	67	38	4

*Adequate was defined as a respiratory specimen obtained 2–14 days after onset of fever.

Of the 12 confirmed cases, 7 were in children <14 years of age, and 5 were in adults (Table 2). Fever was often the first symptom, and dyspnea often occurred a median of 5 days after illness onset (range 1–16). During the initial evaluation at hospital, all patients were found to have fever, cough, and dyspnea, and almost half had myalgia and diarrhea. The hospital course was characterized by intermittent high fevers and persistent cough productive of thick sputum. One patient had a small amount of hemoptysis. Later in the course of the disease, organ failure or dysfunction was commonly observed, including respiratory failure in 9 (75%) patients, cardiac failure in 5 (42%), and renal dysfunction in 4 (33%).

**Table 2:** Characteristics and clinical findings of confirmed avian influenza A (H5N1) cases in Thailand, 2004*

Characteristics	Patient no.												
	1	2	3	4	5	6	7	8	9	10	11	12	%
Age (y), sex	2, M	27, F	31, M	46, F	5, M	6, M	6, M	6, M	7, M	13, M	39, F	58, F	67 (M)
Symptoms													
Fever	+	+	+	+	+	+	+	+	+	+	+	+	100
Rhinorrhea	–	+	–	–	+	+	+	–	–	–	–	–	33
Cough	+	+	+	+	+	+	+	+	+	+	+	+	100
Sore throat	+	+	–	+	+	–	+	+	+	+	–	+	75
Myalgia	–	+	+	+	–	–	–	+	–	–	+	–	42
Dyspnea	+	+	+	+	+	+	+	+	+	+	+	+	100
Diarrhea	+	–	+	–	+	–	–	+	–	–	+	–	42
Abdominal pain	–	–	–	–	+	–	+	–	–	–	–	–	17
Conjunctivitis	–	–	–	–	–	–	–	–	–	–	–	–	0
Vomiting	–	–	–	–	–	–	–	+	–	+	+	–	25
Laboratory values													
Hematocrit (vol%)	30	39	38	46	39	32	39	40	41	37	33	38	
Total leukocyte count	4,200	13,600	4,660	7,360	5,600	1,200	2,200	4,900	4,100	2,000	3,300	5,680	
Total lymphocyte count	2,646	3,400	513	2,429	2,296	624	638	1,763	1,435	580	660	454	
Platelet count (x10^3^)	214	306	171	272	94	89	140	111	304	150	380	185	
Treatment													
Oseltamivir	+	–	+	–	+	+	–	+	+	+	–	–	58
Corticosteroids	+	–	+	–	–	+	+	+	+	+	+	–	67
Outcome													
ARDS	–	–	+	–	+	+	+	+	+	+	+	+	75
Inotropic support	–	–	–	–	+	–	–	+	+	+	–	+	42
Peak AST (U)	129	18	74	NA	70	790	175	280	120	34	394	NA	
Peak ALT (U)	57	23	41	NA	47	150	43	50	52	47	106	NA	
Peak BUN (mg/dL)	NA	8	10.7	NA	12	NA	14	22	10	132	37	39	
Peak creatinine (mg/dL)	NA	0.8	1.07	NA	0.7	NA	1.7	1.1	0.7	8.1	3.6	2.3	
Survival (day of death)	+	+	+	+	– (13)	– (20)	– (18)	– (8)	– (29)	– (16)	– (13)	– (8)	33

*M, male; f, female; +, yes, –, no, NA, not applicable; ARDS, acute respiratory distress syndrome; AST, aspartate aminotransferase; ALT, alanine aminotransferase; BUN, blood urea nitrogen.

Routine laboratory tests on admission showed leukopenia in 7 (58%) patients, lymphopenia in 7 (58%), and thrombocytopenia in 4 (33%) (Table 2). During the course of illness, elevated serum transaminase values were documented in 67% of patients, although they were >8 times normal in only 17%. Serum creatinine rose to >1.5 mg/dL in 4 (33%) patients. Blood cultures were negative in all patients. One adult patient was found to be HIV seropositive, and 1 pediatric patient had a mycoplasma titer of 1:160.

**Figure 2:**
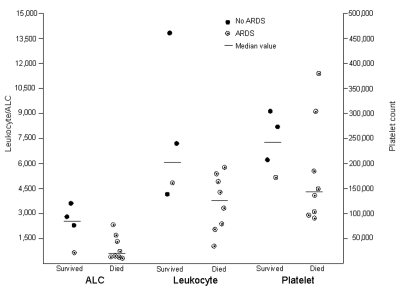
Distribution of the absolute lymphocyte count (ALC), total leukocyte count, and platelet count on admission for 4 patients who survived and 8 who died of human influenza A (H5N1) infection, Thailand, 2004. ARDS, acute respiratory distress syndrome.

Admission leukocyte and platelet counts tended to be more depressed in the 8 patients who died than in the 4 patients who survived (Figure 2). ARDS was associated with a fatal outcome (p = 0.02), and depressed admission leukocyte and platelet counts were also associated with ARDS development. The most pronounced difference was in the absolute lymphocyte count, with a mean of 995 in those with ARDS vs. 2,825 in those without (p = 0.002). A low absolute lymphocyte count on admission was also associated with death (mean of 1,056/mm^3^ in those who died compared to 2,247/mm^3^ in those who survived, p = 0.05). In addition, the median total leukocyte count was 3,700/mm^3^ for those who died compared with 6,010/mm^3^ for those who survived (p = 0.09), and the median platelet count was 145,000/mm^3^ in those who died and 243,000/mm^3^ in those who survived (p = 0.17).

**Figure 3:**
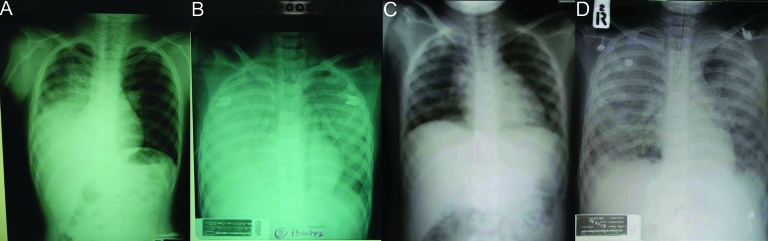
Chest radiographs from patients 8 and 9. Panel A demonstrates patchy alveolar infiltration of the right lower lung on day 5 of illness for patient 9; panel B demonstrates the progression to acute respiratory disease syndrome (ARDS) on day 8. Panel C shows interstitial infiltration of both lungs of patient 8 on day 4 of illness; panel D shows the rapid progression to ARDS by day 6.

All 12 patients had abnormal chest radiographs a median of 7 days after onset of fever (range 3–17 days). Two patients had interstitial infiltration, and 10 had patchy lobar infiltrates in a variety of patterns (single lobe, multiple lobes, unilateral or bilateral distributions). The radiographic pattern progressed to diffuse bilateral ground-glass appearance, with clinical features compatible with ARDS, in all 8 patients who died and in 1 patient who survived (Figure 3). A pneumothorax developed in l patient during mechanical ventilation. The median time from onset to ARDS development was 6 days (range 4–13).

**Figure 4:**
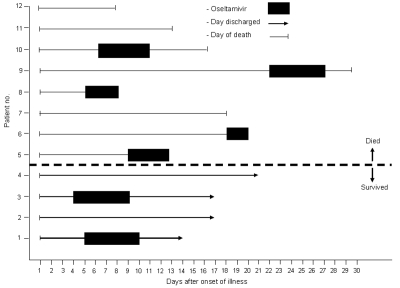
Timing of the clinical course and oseltamivir treatment for 4 patients who survived and 8 patients who died of human influenza A (H5N1) infection, Thailand, 2004.

Treatment for all patients included broad-spectrum antimicrobial drugs aiming to cover most of the usual and unusual respiratory pathogens. Eight patients were treated with corticosteroid drugs, including 2 patients who survived and 6 patients who died. Seven patients were treated with the neuraminidase inhibitor oseltamivir at various stages of illness. Treatment tended to have been started earlier in those who survived (a median of 4.5 days from onset compared with 9 days for those who died), and both survivors who were treated received the complete 5-day course of drug, whereas 2 of 5 patients who died received the complete 5-day course (Figure 4).

**Figure 5:**
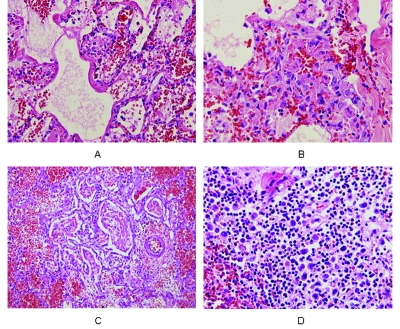
Pathologic findings from a patient (number 6) with confirmed influenza A (H5N1) infection. All slides are stained with hematoxylin and eosin, shown at 40x objective. Panel A shows hyaline membrane formation lining the alveolar spaces of the lung and vascular congestion with a few infiltrating lymphocytes in the interstitial areas. Reactive fibroblasts are also present. Panel B is an area of lung with proliferating reactive fibroblasts within the interstitial areas. Few lymphocytes are seen, and no viral intranuclear inclusions are visible. Panel C shows fibrinous exudates filling the alveolar spaces, with organizing formation and few hyaline membranes. The surrounding alveolar spaces contain hemorrhage. Panel D is from a section of spleen, showing numerous atypical lymphoid cells scattered around the white pulp. No viral intranuclear inclusions are seen.

Pathologic tissues from the lungs and spleen of 3 patients were available for analysis in the current report. A fourth patient (number 6) was autopsied but is the subject of a separate report. The lungs showed diffuse alveolar damage, with hyaline membrane formation, reactive fibroblasts, and areas of hemorrhage. The spleen had numerous atypical lymphocytes but no viral inclusions (Figure 5).

All 12 confirmed patients resided in a village with abnormal chicken deaths (Table 3). Nine lived in a house whose backyard chickens died unexpectedly. Direct contact with dead chickens was reported in 8 patients, with a median of 4 days between the last exposure and the onset of symptoms (range 2–8 days). The details of exposures in these case-patients and in groups of matched controls are the subject of a separate investigation.

**Table 3:** Brief history of exposure of the 12 confirmed case-patients

Patient no.	Province/sex/age (y)	Exposure history
1	Supanburi/M/2	Raised chickens in backyard. Chickens died unexpectedly 5 days before illness onset. Frequently played with chickens and had direct contact with carcasses.
2	Uttradit/F/27	Raised chickens in backyard, but chickens did not die. Two months before onset, ducks in a nearby area died unexpectedly.
3	Nakornratchasima/M/31	Raised chickens in backyard. Three days before onset, chickens started to die. The last patient died on the date he became sick. He buried all carcasses.
4	Lopburi/F/46	Raised 60 chickens in back yard. All chickens died unexpectedly 1 month before onset. She burned and buried carcasses without protection.
5	Khonkaen/M/5	Raised fighting cocks that died 4 days before onset. Reported direct contact with carcasses. Ate chicken with suspected H5N1 influenza.
6	Kanchanaburi/M/6	No poultry in family. Helped slaughter one ill chicken 2 days before onset.
7	Sukhothai/M/6	Mother slaughtered 2 ill chickens in house 4 days before onset. No direct contact with chickens. Mother got sick on same day and died without laboratory confirmation.
8	Kanchanaburi/M/6	Chickens in backyard died unexpectedly. Grandfather slaughtered ill chickens. No direct contact with chickens but played near slaughtering area.
9	Supanburi/M/7	No poultry in family. Frequently played on ground near a chicken farm that reported unexpected poultry deaths.
10	Chaiyapoum/M/13	Helped raise chickens in backyard. Eight days before onset, chickens died unexpectedly and patient assisted with slaughtering.
11	Patumthani/F/39	Factory worker living in province A during weekdays but in province B on weekends. Fighting cocks lived at a neighboring house. Province B reported outbreaks 2 months before onset. No contact with live or dead chickens.
12	Supanburi/F/58	Raised 40–50 chickens in backyard. Chickens started to die 5 days before onset. Buried and slaughtered ill chickens every day until onset date.

## Discussion

The detection of a few human infections with influenza A (H5N1) in the context of an avian epizootic involving at least 8 countries has proven to be a considerable challenge. The history of direct contact with sick and dying poultry, young age of many patients, pneumonia and lymphopenia, and progression to ARDS in spite of broad-spectrum antimicrobial treatment indicate that specific laboratory testing for H5 influenza should be sought. Ideally, such information should be routinely collected and used to minimize opportunities for recombination of this virulent new pathogen with existing human influenza viruses.

The optimal treatment for case-patients with suspected H5 infection is not known, but in vitro susceptibility testing suggests that resistance to adamantanes is a common feature of H5 isolates from 2004 (*11*), whereas these isolates remain susceptible to the neuraminidase inhibitors. Although no controlled data are available on which to base treatment recommendations, our observations were that the 4 patients who survived tended to have been treated with oseltamivir earlier in the course of their disease. We advocate using this agent in the early treatment of case-patients with suspected H5N1 influenza, in agreement with the recommendations of WHO (*16*). Controlled trials of oseltamivir and corticosteroid treatment would be helpful in confirming or refuting any specific benefit.

Approximately 1,820,387,000 persons live in the 8 countries in Asia that reported poultry epidemics with avian influenza A (H5N1) in 2004 (≈30% of the world's population). One community survey in Thailand found that 12%–61% of rural residents had regular contact with backyard birds (*17*). Thus, the 12 cases we report likely represent the end result of hundreds of thousands of potential exposures and an unknown number of human cases. Perhaps in part because few distinctive features of human disease caused by avian influenza have been reported, and specific diagnostic tests for H5 disease are not widely available, human cases have been few and have been reported only from Vietnam and Thailand.

Among >600 possible case-patients reported to the Thai Ministry of Public Health, most reported clear exposure to sick poultry, and the demographic characteristics were similar among confirmed, suspected, and excluded groups. All confirmed patients had an adequate specimen submitted and processed, whereas 10% of the suspected patients and 19% of those excluded had inadequate specimens. The availability of properly collected specimens and use of specific laboratory tests for influenza A (H5N1) will be essential for monitoring the ongoing risk from this pathogen in East Asia.

Human infections with highly pathogenic avian influenza may be easy to miss in the context of the regular incidence of pneumonia in much of rural Asia, where the capacity to make specific etiologic diagnoses remains limited. We found certain features to be helpful, as have investigators in Vietnam (*9*). Eight of the 12 patients had direct exposures to ill poultry 2–8 days before onset. Seven of the 12 were young children, and routine laboratory testing at the time of admission to hospital identified marked lymphopenia in 8. Although the initial chest radiographs would not immediately identify these cases as unusual, deaths in children and younger adults from hospitalized, radiographically confirmed pneumonia typically range from 1% to 10% and from 1%–5% among patients with radiographically confirmed pneumonia in rural Thailand (*18**–**20*). Thus, the progression in 9 of the 12 patients to ARDS, followed by the death of 8 patients, separates these cases as a form of unusually severe pneumonia.

The disease may in fact be more severe than that seen in Hong Kong in 1997. Of the 34 cases officially reported to the WHO in 2004, 23 (68%) patients died compared to 6 (33%) of those in Hong Kong (p = 0.02). Several lines of evidence indicate that the H5N1 viruses have evolved to more virulent forms since 1997, with different antigenic structure (*21*), internal gene constellations (*22*), and an expanded host range (*23**,**24*). This virologic evolution may be a factor in the persistence of H5N1 viruses in the avian populations. Since the 1997 outbreak, Hong Kong has experienced a series of reintroductions of H5 viruses, despite instituting unusually stringent control measures, including the culling of all poultry in the territory, strict regulations of live poultry markets, and monthly "off days," in which all markets are emptied and cleaned (*22**,**25*). H5 outbreaks in poultry have also recurred repeatedly in Thailand, Vietnam, and elsewhere despite intensive control measures (*26*), and recurrences should be anticipated for the foreseeable future.

If H5 viruses do persist, they will likely continue to evolve, potentially to forms more easily transmitted from person to person. We identified no suspected or confirmed cases among Thai health personnel, supporting the experience from Vietnam and Hong Kong that efficient human-to-human transmission has not occurred (*9**,**27*). Serologic studies of healthcare workers and household contacts of patients in the 1997 Hong Kong outbreak provided evidence of occasional seroconversions associated with close exposures. These findings indicate that inefficient transmission is possible and reinforce the importance of infection control precautions (*28**,**29*). Studies of healthcare workers and poultry cullers in Thailand are under way to determine whether similar seroconversions may have occurred after exposure to patients with the 2004 viruses.

**Figure 6:**
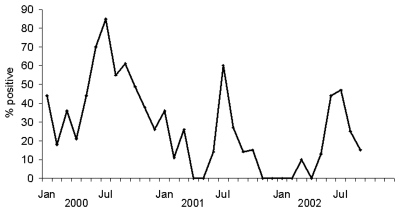
Seasonal variation in viral isolations of human influenza A (H3N2), A (H1N1), and B, in Thailand.

In addition to gradual mutational changes, H5 viruses have the potential to reassort with existing human influenza viruses to produce a strain with high virulence and efficient transmissibility. In this context, the known pattern of human influenza isolations in Thailand raises particular concerns about control of avian influenza during the months from June to August, when human influenza can be expected to peak (Figure 6).

After the official announcement of the first human case on January 23, a national public education campaign was carried out through the mass media and thousands of village health volunteers. Villagers, especially children, were informed to avoid exposure to ill poultry. According to the Department of Livestock, ≈40 million chickens in 160 affected villages of 41 provinces were slaughtered from January to May 2004. Within 2 months of implementing widespread poultry culling, quarantine measures, and the public education campaign, the number of potential cases reported to the surveillance system decreased dramatically and confirmed human cases ceased, despite interim improvement in the quality of surveillance and laboratory testing. The course of this outbreak reconfirms observations from the smaller 1997 outbreak in Hong Kong that early detection of human cases and aggressive public health and agricultural interventions can save lives (*30*).

We believe this outbreak of H5N1 is unlikely to be the last because of the formidable challenges in eradicating the virus, and the potential reservoir in waterfowl (*31*). We must be well prepared for a future surge of either small or large outbreaks, early detection must be ensured, information shared, and control measures for both animals and humans promptly implemented.
